# Intramuscular sex steroid hormones are associated with skeletal muscle strength and power in women with different hormonal status

**DOI:** 10.1111/acel.12309

**Published:** 2015-01-20

**Authors:** Eija Pöllänen, Reeta Kangas, Mia Horttanainen, Paula Niskala, Jaakko Kaprio, Gillian Butler-Browne, Vincent Mouly, Sarianna Sipilä, Vuokko Kovanen

**Affiliations:** 1 Department of Health Sciences, Gerontology Research Center, University of JyväskyläJyväskylä, Finland; 2 Department of Public Health, University of HelsinkiHelsinki, Finland; 3 Institute for Molecular Medicine, University of HelsinkiHelsinki, Finland; 4 National Institute for Health and WelfareHelsinki, Finland; 5 Myology Research Center, Institut de Myologie, Sorbonne Universités, UPMC University Paris 06, UMR974, INSERM U974, CNRS FRE 3617F-75013, Paris, France

**Keywords:** intracrine organ, steroidogenesis, muscle performance, local hormone synthesis, muscle steroids

## Abstract

Estrogen (E_2_)-responsive peripheral tissues, such as skeletal muscle, may suffer from hormone deficiency after menopause potentially contributing to the aging of muscle. However, recently E_2_ was shown to be synthesized by muscle and its systemic and intramuscular hormone levels are unequal. The objective of the study was to examine the association between intramuscular steroid hormones and muscle characteristics in premenopausal women (*n* = 8) and in postmenopausal monozygotic twin sister pairs (*n* = 16 co-twins from eight pairs) discordant for the use of E_2_-based hormone replacement. Isometric skeletal muscle strength was assessed by measuring knee extension strength. Explosive lower body muscle power was assessed as vertical jump height. Due to sequential nature of enzymatic conversion of biologically inactive dehydroepiandrosterone (DHEA) to testosterone (T) and subsequently to E_2_ or dihydrotestosterone (DHT), separate linear regression models were used to estimate the association of each hormone with muscle characteristics. Intramuscular E_2_, T, DHT, and DHEA proved to be significant, independent predictors of strength and power explaining 59–64% of the variation in knee extension strength and 80–83% of the variation of vertical jumping height in women (*P* < 0.005 for all models). The models were adjusted for age, systemic E_2_, and total body fat mass. The statistics used took into account the lack of statistical independence of twin sisters. Furthermore, muscle cells were shown to take up and actively synthesize hormones. Present study suggests intramuscular sex steroids to associate with strength and power regulation in female muscle providing novel insight to the field of muscle aging.

## Introduction

Aging, in general, is associated with gradual decrements in tissue and organ functions. In women, menopause characterized by the loss of ovarian function represents an aging process that leads to changes in the systemic steroid hormone profile from a regularly fluctuating estrogen cycle to very low and constant levels. This decrease in systemic estrogen may, however, have tissue-specific effects on estrogen-responsive tissues such as skeletal muscle (Wend *et al*., 2012). Premenopausal women tend to have better muscle properties than postmenopausal women while the use of postmenopausal hormone replacement therapy (HRT) partially prevents aging-related decrements or even improves muscle function during the early years of menopause (Sipilä *et al*., 2001; Lowe *et al*., 2010). We have previously shown that the use of HRT, which increases systemic estrogen levels, helps to maintain better body and muscle composition and function by influencing muscle gene expression (Pöllänen *et al*., 2010; Ronkainen *et al*., 2010). However, systemic estrogen concentration measured in the serum cannot be directly taken as an estimate of the hormone content of the tissue (Pöllänen *et al*., 2011). It is generally accepted that aromatization of testosterone (T) to 17β-estradiol (E_2_) by the aromatase cytochrome P450 enzyme (called aromatase here after) occurs in skeletal muscle (Longcope *et al*., 1978). More recently, Aizawa *et al*. (2007) showed that rat muscle cells are able to take up the precursor hormone dehydroepiandrosterone (DHEA) and use it to synthesize biologically active sex steroid hormones: T and E_2_. They also showed that acute exercise and endurance training induces steroidogenesis in rat skeletal muscle (Aizawa *et al*., 2010, 2011) while Vingren *et al*. (2008) found no differences in human muscular steroidogenesis in response to a single bout of resistance exercise. Recently, Sato *et al*. (2014a) showed that resistance training restores muscle androgen levels in older men. Our previous study showed differences in systemic and intramuscular hormone concentrations parallel to differences in thigh muscle size, performance, and quality in healthy pre- and postmenopausal women not using HRT (Pöllänen *et al*., 2011). Currently, there are no studies which have investigated the effects of intramuscular steroid hormone levels or local steroidogenesis on muscle properties or performance in women with different hormonal status. The aim of this study was to investigate the associations between intramuscular sex steroid hormones and muscle properties in different menopausal backgrounds. The unique design with postmenopausal HRT discordant twin pairs reinforced with additional group of premenopausal women was used. In addition, this current study investigates *in vitro* the ability of muscle cells to take up and synthesize sex steroid hormones and to transcriptionally respond to hormonal stimuli.

## Results

### Premenopausal women have better muscle properties than postmenopausal women

This study is a cross-sectional co-twin case-controlled study using postmenopausal monozygotic (MZ) twin pairs (*n* = 16 co-twins from eight pairs) with additional premenopausal control group (*n* = 8). Premenopausal women (aged 32 ± 2 years) did not use hormonal preparations (Pöllänen *et al*., 2011). Postmenopausal MZ twin pairs (aged 58 ± 2 years) were discordant for the use of E_2_-based HRT (Ronkainen *et al*., 2009). Characteristics of the study participants are presented in the Table1. There were no differences in anthropometry, body composition or the level of physical activity between the study groups. However, premenopausal women were stronger and more powerful and had larger muscles than postmenopausal women. Premenopausal women's knee extension strength was 31.0% higher compared to HRT users and 33.5% higher compared to nonusers (*P *=* *0.008 and 0.010, respectively). According to vertical jumping height measurement, premenopausal women had 60.0% higher muscle power than postmenopausal HRT users (*P *=* *0.001) and 95.5% higher power than postmenopausal nonusers (*P *<* *0.001). In addition, premenopausal women had 9.5% larger thigh muscle cross-sectional area (CSA) than postmenopausal HRT users (not significant difference) and 15.1% larger thigh CSA than nonusers (*P *=* *0.034). Postmenopausal HRT users had significantly greater muscle power than their nonusing co-sisters (22.2%, *P *=* *0.006).

**Table 1:** Characteristics of the study participants

	Premenopausal women (*n* = 8)	Postmenopausal HRT users (*n* = 8)	Postmenopausal nonusers (*n* = 8)	*P*-values		
				Pre- vs. HRT users	Pre- vs. nonusers	HRT vs. nonusers
Age (years)	32 ± 2	58 ± 2	58 ± 2			
MET index	4.8 ± 3.5	8.4 ± 8.1	4.4 ± 2.4	0.271	0.799	0.142
Body composition						
Height (cm)	164.6 ± 3.7	162.6 ± 4.5	162.1 ± 3.8	0.350	0.208	0.240
Weight (kg)	69.5 ± 10.7	66.3 ± 8.7	69.8 ± 14.1	0.522	0.971	0.307
LBM (kg)	45.5 ± 3.5	43.5 ± 3.6	42.8 ± 4.6	0.277	0.201	0.518
Fat mass (kg)	21.3 ± 8.3	19.9 ± 6.0	24.1 ± 9.5	0.696	0.552	0.066
Muscle performance						
KES (N)	584.2 ± 106.8	446.1 ± 66.3	437.7 ± 90.6	**0.008**	**0.010**	0.685
VjH (cm)	26.4 ± 6.1	16.5 ± 3.0	13.5 ± 4.4	**0.001**	**<0.001**	**0.006**
Thigh CSA (cm^2^)	106.1 ± 11.4	96.9 ± 12.1	92.2 ± 12.2	0.143	**0.034**	0.171
Systemic variables						
LH (IU L^−1^)	12.8 ± 12.6	25.4 ± 5.0	28.6 ± 6.0	**0.020**	**0.006**	0.273
FSH (IU L^−1^)	6.2 ± 2.7	66.3 ± 24.3	95.6 ± 11.7	**<0.001**	**<0.001**	**0.010**
SHBG (nmol L^−1^)	49.3 ± 16.8	72.3 ± 30.7	47.1 ± 13.1	0.086	0.777	**0.019**

FSH, follicle-stimulating hormone; KES, knee extension strength; LH, luteinizing hormone; LMB, lean body mass; MET index, metabolic equivalent index; SHBG, serum hormone-binding globulin; Thigh CSA, cross-sectional area of lean thigh muscle compartment; VjH, vertical jumping height.

Statistically significant *P*-values are highlighted by bolding the letters.

### Intramuscular hormone concentrations and systemic hormone levels are different in pre- and postmenopausal women

**Figure 1:**
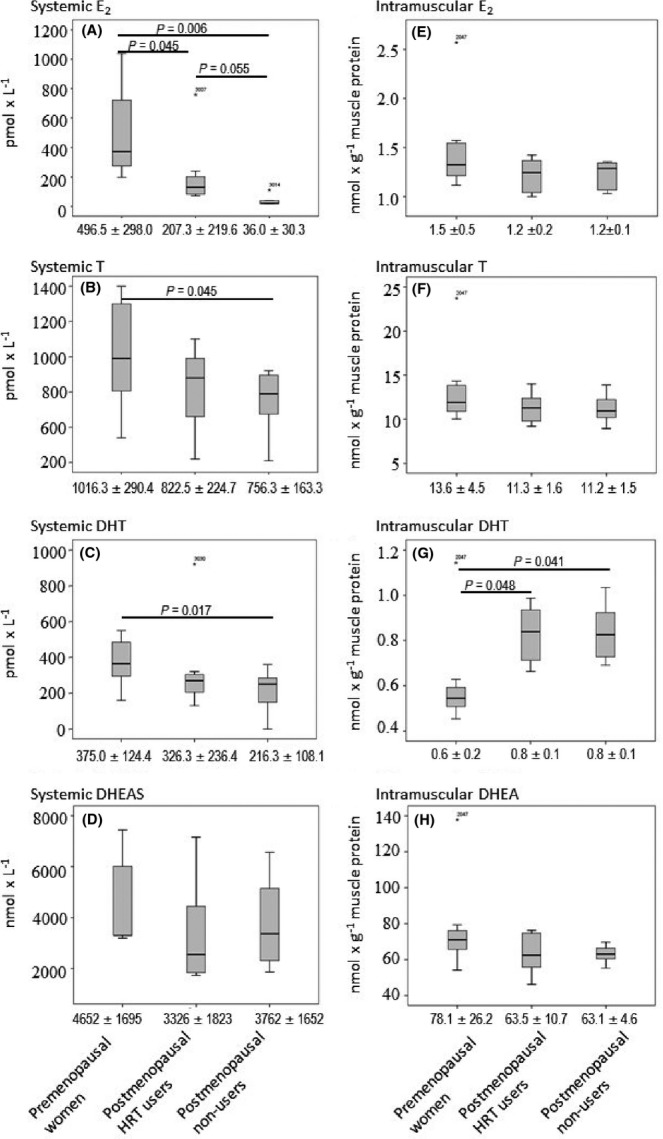
Median and distribution of systemic (A, B, C, D) and intramuscular (E, F, G, H) sex steroid hormone concentrations in premenopausal women and in postmenopausal HRT using MZ twins and their nonusing co-sisters. For each box, the bold central line indicates the group median. The top and bottom edges of each box indicate the upper and lower quartiles, respectively. T-bars that extend from the boxes indicate highest and lowest observations, with the exception of outliers marked with asterisk and ID of the participant. Under each figure, the mean values±standard deviation for each group is presented. E_2_, 17β-estradiol; T, testosterone; DHT, dihydrotestosterone; DHEAS, dehydroepiandrosterone sulfate; DHEA, dehydroepiandrosterone.

Results regarding systemic and intramuscular sex steroid hormones are presented in Fig.1, and results concerning other relevant systemic hormones are summarized in Table1. Premenopausal women had significantly higher systemic E_2_ concentration than postmenopausal HRT users (*P *=* *0.045) and nonusers (*P *=* *0.006), while the difference between HRT users and nonusers was at the borderline of significance (*P *=* *0.055). Systemic concentrations of T and dihydrotestosterone (DHT) were significantly higher in premenopausal as compared to postmenopausal nonusers (*P *=* *0.045 and 0.017, respectively), but there were no significant differences when premenopausal women were compared to postmenopausal HRT users or when HRT users were compared to nonusing co-sisters. No significant differences were observed in systemic sulfated DHEA (DHEAS) concentration between any of the group comparisons. The hormone concentrations measured from muscle samples (Fig.1E–H) did not, however, reflect the systemic concentrations (Fig.1A–D). There were significant differences between the groups only for intramuscular DHT (Fig.1G) that was lower in premenopausal women when compared to both postmenopausal groups (*P *=* *0.048 for HRT users and *P *=* *0.041 for nonusers). Surprisingly, there were virtually no differences between groups in the intramuscular E_2_ and T although systemic differences, especially for E_2_, were observed. In particular, the use of E_2_-containing HRT did not affect the intramuscular E_2_ concentration in the HRT discordant MZ twins. Furthermore, there were strong intraclass correlation between co-sisters regarding the intramuscular concentrations of E_2_ (*r* = 0.931, *P *=* *0.001), DHT (*r* = 0.818, *P *=* *0.013), and DHEA (*r* = 0.765, *P *=* *0.028), suggesting that intramuscular steroidogenesis is more subject to genetic than systemic control. One premenopausal participant had exceptionally high hormone concentrations in her muscle sample (Fig.1E–H). Excluding this outlier from the statistical testing did not change the significance of the results presented above except that differences between the premenopausal group and the postmenopausal nonusers and HRT users were stronger (*P *<* *0.001 for both comparisons). Therefore, we did not exclude this participant from any of the analyses performed in this study.

### Intramuscular sex steroid levels are associated with muscle performance

Linear regression analyses were performed to evaluate the association between intramuscular sex steroid hormones and muscle characteristics (Table3 and S1). As the synthesis of steroid hormones proceeds sequentially from the biologically inactive precursor DHEA to T and further to E_2_ or DHT, separate models were developed for each hormone. According to the raw models, intramuscular E_2_, T, and DHEA, but not DHT, were significantly associated with muscle strength and power, explaining 36–39% of the variation in knee extension strength (Table2) and 37–45% of the variation in vertical jumping height (Table3). Intramuscular hormones were not associated with thigh muscle CSA (Table S1, Supporting information). To control for possible confounders, models were adjusted for age, systemic E_2_, and total body fat mass. According to the adjusted models, all intramuscular hormones were significantly associated with muscle strength and power (for all hormones *P *<* *0.005). Intramuscular E_2_ explained 63%, intramuscular T 64%, intramuscular DHT 64%, and intramuscular DHEA 59% of the variation in knee extension strength. From the variation of muscle power, intramuscular E_2_ explained 83%, intramuscular T 83%, intramuscular DHT 80%, and intramuscular DHEA 83%.

**Table 2:** Linear regression models for skeletal muscle strength (knee extension strength)

	Model 1 B (SE)	Model 2 B (SE)	Model 3 B (SE)	Model 4 B (SE)
Estrogen models				
Intramuscular E_2_	204.9 (54.6)	153.1 (52.5)	156.3 (55.1)	189.4 (53.5)
	P = 0.002	P = 0.011	P = 0.013	P = 0.003
Age		−3.5 (1.6)	−4.1 (1.4)	−3.6 (1.3)
		P = 0.044	P = 0.012	P = 0.013
Systemic E_2_			−0.04 (0.04)	0.01 (0.02)
			P = 0.266	P = 0.508
Fat mass				5.7 (1.6)
				P = 0.003
*R*^2^ for a model	0.362	0.493	0.500	0.628
Testosterone models				
Intramuscular T	22.6 (5.9)	17.8 (5.9)	18.0 (6.2)	19.6 (5.6)
	P = 0.002	P = 0.010	P = 0.012	P = 0.004
Age		−3.6 (1.5)	−4.1 (1.4)	−3.8 (1.3)
		P = 0.029	P = 0.010	P = 0.009
Systemic E_2_			−0.04 (0.03)	0.02 (0.02)
			P = 0.309	P = 0.515
Fat mass				4.8 (1.4)
				P = 0.005
*R*^2^ for a model	0.389	0.535	0.540	0.637
DHT models				
Intramuscular DHT	36.4 (145.3)	335.9 (100.7)	335.9 (101.9)	360.1 (90.0)
	P = 0.806	P = 0.005	P = 0.005	P = 0.001
Age		−8.0 (1.7)	−8.3 (1.7)	−8.4 (1.6)
		P < 0.001	P < 0.001	P < 0.001
Systemic E_2_			−0.02 (0.03)	0.03 (0.02)
			P = 0.555	P = 0.158
Fat mass				4.7 (1.4)
				P = 0.005
*R*^2^ for a model	0.004	0.544	0.545	0.635
DHEA models				
Intramuscular DHEA	3.8 (0.9)	2.9 (0.9)	2.9 (0.9)	3.0 (0.8)
	P = 0.001	P = 0.006	P = 0.008	P = 0.002
Age		−3.6 (1.7)	−4.1 (1.5)	−3.9 (1.5)
		P = 0.049	P = 0.018	P = 0.019
Systemic E_2_			−0.1 (0.1)	0.01 (0.02)
			P = 0.311	P = 0.788
Fat mass				4.1 (1.8)
				P = 0.034
*R*^2^ for a model	0.372	0.508	0.514	0.586

**Table 3:** Linear regression models for skeletal muscle power (vertical jumping height)

	Model 1 B (SE)	Model 2 B (SE)	Model 3 B (SE)	Model 4 B (SE)
Estrogen models				
Intramuscular E_2_	15.6 (3.1)	10.4 (2.3)	9.9 (1.6)	8.4 (1.6)
	P < 0.001	P < 0.001	P < 0.001	P < 0.001
Age		−0.4 (0.1)	−0.3 (0.1)	−0.3 (0.1)
		P < 0.001	P = 0.001	P = 0.002
Systemic E_2_			0.01 (0.01)	0.01 (0.01)
			P = 0.014	P = 0.056
Fat mass				−0.3 (0.1)
				P < 0.001
*R*^2^ for a model	0.454	0.739	0.772	0.830
Testosterone models				
Intramuscular T	1.5 (0.3)	1.0 (0.2)	1.0 (0.2)	0.8 (0.2)
	P < 0.001	P < 0.001	P < 0.001	P < 0.001
Age		−0.4 (0.1)	−0.3 (0.1)	−0.3 (0.1)
		P < 0.001	P = 0.001	P = 0.001
Systemic E_2_			0.01 (0.01)	0.01 (0.01)
			P = 0.011	P = 0.064
Fat mass				−0.3 (0.05)
				P < 0.001
*R*^2^ for a model	0.367	0.709	0.749	0.828
DHT models				
Intramuscular DHT	−7.0 (11.8)	15.1 (4.8)	15.1 (4.6)	13.5 (0.1)
	P = 0.563	P = 0.007	P = 0.005	P < 0.001
Age		−0.6 (0.1)	−0.5 (0.1)	−0.5 (0.1)
		P < 0.001	P < 0.001	P < 0.001
Systemic E_2_			0.01 (0.01)	0.01 (0.01)
			P = 0.007	P = 0.044
Fat mass				−0.3 (0.1)
				P < 0.001
*R*^2^ for a model	0.031	0.664	0.714	0.802
DHEA models				
Intramuscular DHEA	0.3 (0.1)	0.2 (0.1)	0.2 (0.2)	0.2 (0.2)
	P < 0.001	P < 0.001	P < 0.001	P < 0.001
Age		−0.4 (0.1)	−0.3 (0.1)	−0.3 (0.1)
		P = 0.001	P = 0.003	P = 0.003
Systemic E_2_			0.01 (0.01)	0.01 (0.01)
			P = 0.014	P = 0.068
Fat mass				−0.3 (0.1)
				P < 0.001
*R*^2^ for a model	0.371	0.696	0.734	0.831

### Gene expression of aromatase and steroid hormone receptors during *in vitro* differentiation of myoblasts

**Figure 2:**
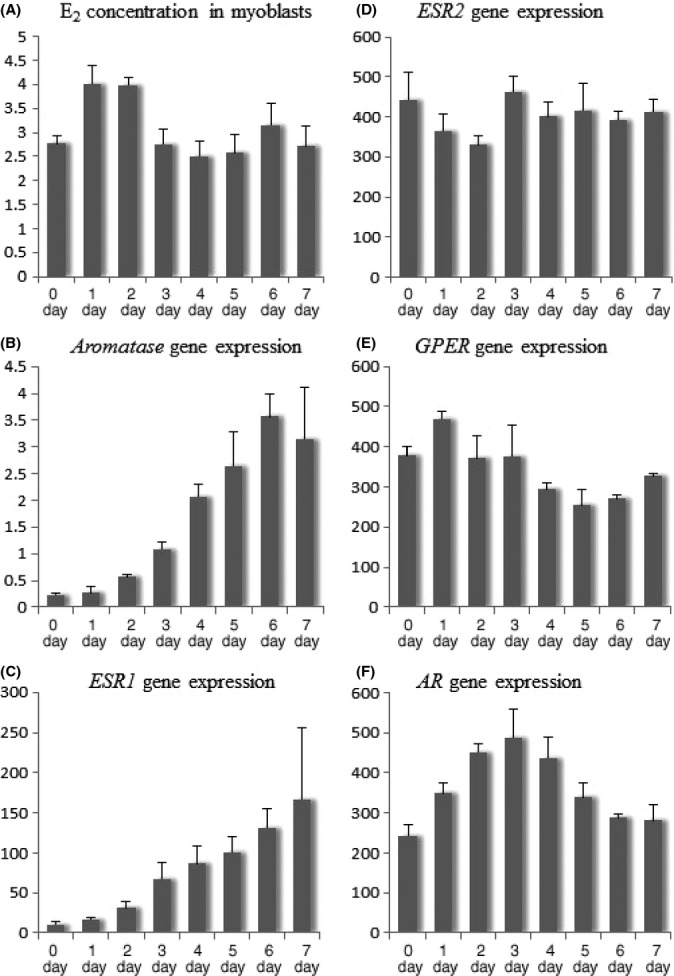
E_2_ concentration (A) and gene expressions (B, C, D, E, F) during differentiation of mononucleated myoblasts into multinucleated myotubes. Gene expressions are normalized to the expression of *GAPDH* and presented in arbitrary units. E_2_, 17β-estradiol; *ESR1*, estrogen receptor 1; *ESR2*, estrogen receptor 2; *GPER*, G-protein-coupled estrogen receptor 1; *AR*, androgen receptor.

To assess whether human muscle cells have a potential to synthesize E_2_ and to activate hormone receptor-dependent signaling, the *in vitro* expression of *aromatase* (*CYP19A1* gene), the enzyme converting T to E_2_, and steroid hormone receptors, including *ESR1*,*ESR2*,*GPER,* and androgen receptor (*AR*), were measured. Human muscle progenitor cells (myoblasts) were induced to differentiate to form multinucleated myotubes. Differentiating cells were collected at 24-h intervals. At day 3, multinucleated myotubes are clearly visible and differentiation continues thereafter. Interestingly, similar levels of E_2_ were detected in the myoblasts at day 0 before switching to differentiation media and throughout the following 7 days of differentiation (Fig.2A). The expression of measured genes was readily detectable at day 0 in the proliferating myoblasts (Fig.2). However, for the first few days, the expression of *aromatase* (Fig.2B) and *ESR1* (Fig.2C) was quite low and then increasing steadily in the myotubes during differentiation of myotubes. The peak of expression was observed at days 6–7, while the expression of *AR* peaked already at day 3 (Fig.2F).

### Pre- and postmenopausal female sera modify the gene expression of aromatase and steroid hormone receptors

**Figure 3:**
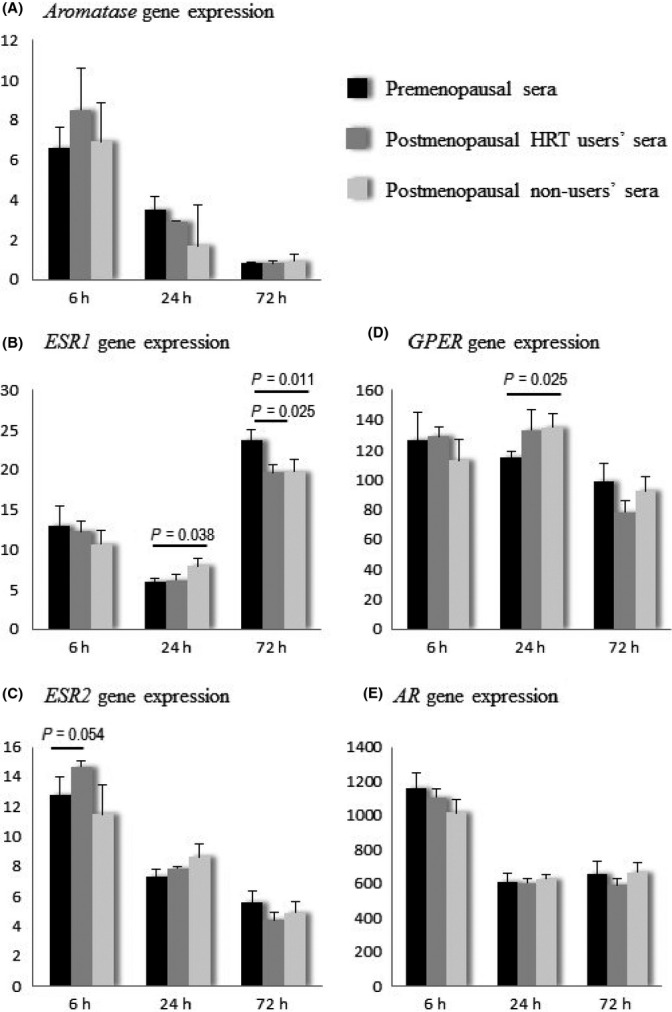
The gene expressions of *aromatase* (A) and sex steroid hormone receptors (B, C, D, E) in myotubes exposed to female serum. Gene expressions are normalized to the expression of *GAPDH* and presented in arbitrary units. *ESR1*, estrogen receptor 1; *ESR2*, estrogen receptor 2; *GPER*, G-protein-coupled estrogen receptor 1; *AR*, androgen receptor.

After verifying that myogenic cells in our *in vitro* model are able to produce E_2_ and its receptors, we used the same model to investigate whether these cells are responsive to female sera with different hormonal content. Multinucleated muscle cells (5 day myotubes) were exposed to pre- and postmenopausal serum obtained from the same study participants who were characterized previously (Fig.1 and Table1). At day 5, half of the differentiation medium was changed to differentiation medium supplemented with premenopausal serum, postmenopausal serum from nonusers, or postmenopausal serum from HRT users. Each serum pool contained equal amounts of serum from the eight women of the respective group and was applied to the cells in differentiation medium to deliver 10% v/v final serum concentration. To detect both acute and later effects, serum-exposed cells were collected 6, 24, and 72 h after exposure. The prolonged exposure to all serum pools reduced the expressions of *aromatase*,*ESR2*,*GPER,* and *AR* and increased the expression of *ESR1* (ANOVA *P *<* *0.001 for all comparisons) (Fig.3). There were no significant differences in the expression of *aromatase* (Fig.3A) and *AR* (Fig.3E) in the myotubes exposed to different serum pools at any of the tested time points. At the 6-h time point, only *ESR2* showed indication for group differences being somewhat higher in the myotubes exposed to serum from postmenopausal HRT users than to serum from premenopausal women (*P *=* *0.054, Fig.3C). At 24 h, the expression of *ESR1* was lower in the myotubes exposed to premenopausal serum compared to the postmenopausal nonuser serum exposure (*P *=* *0.038), while at 72 h, the expression was lower for both comparisons (pre- vs. nonuser *P *=* *0.025, pre- vs. HRT user *P *=* *0.011, Fig.3B). At the 24-h time point, the expression of *GPER* was also lower in the myotubes exposed to the premenopausal serum compared to the postmenopausal nonuser and HRT user serum exposure, but only the first comparison remained statistically significant (*P *=* *0.025, Fig.3D).

### The take up of 17β-estradiol does not induce transcriptional changes in steroid hormone signaling-related genes in myotubes

**Figure 4:**
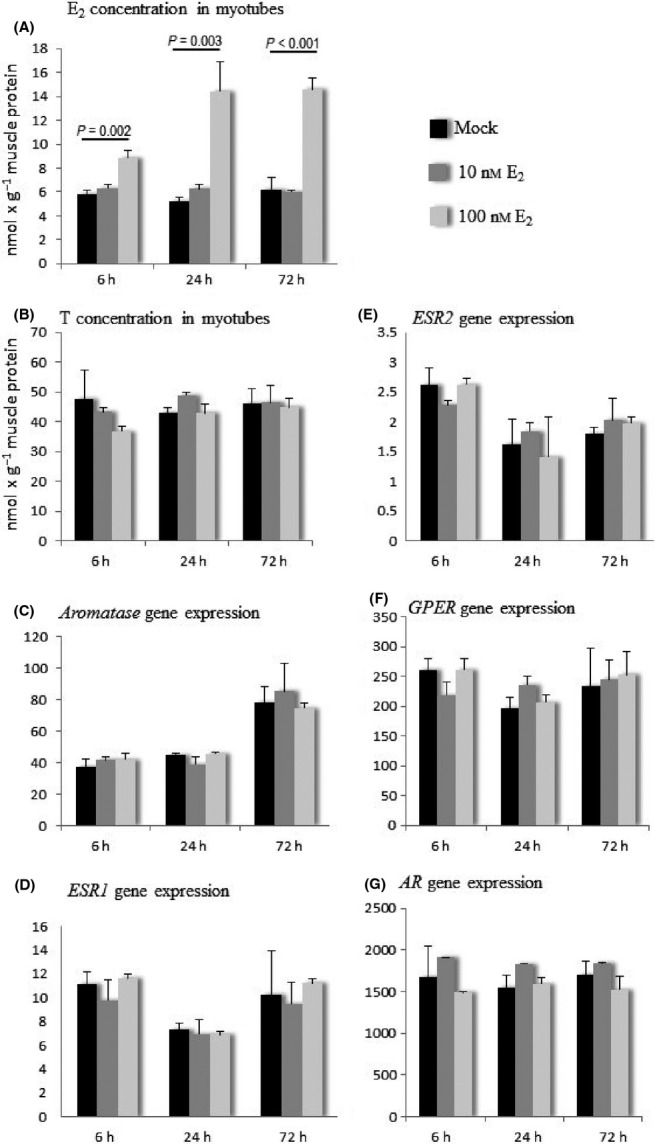
E_2_ (A) and T (B) concentration and the gene expression of *aromatase* (C) and steroid hormone receptors (D, E, F, G) in myotubes exposed to E_2_. Gene expressions are normalized to the expression of *GAPDH* and presented in arbitrary units. E_2_, 17β-estradiol; T, testosterone; *ESR1*, estrogen receptor 1; *ESR2*, estrogen receptor 2; *GPER*, G-protein-coupled estrogen receptor 1; *AR*, androgen receptor.

To investigate whether E_2_ alone is capable of inducing transcriptional changes observed after exposure to the serum samples with different E_2_ concentrations (without possible confounding factors present in human serum), myotubes were exposed to 10 nm E_2_, 100 nm E_2_ or mock, which controls the possible effects of ethanol used as a solvent for E_2_. As with serum exposure, the experiments were started after 5 days of differentiation. Myotubes were harvested after 6-, 24- and 72-h exposure to E_2_. Cells readily took up E_2_ as shown by the increase in the cellular concentration of E_2_ (Fig.4A) and the parallel decrease in the medium concentration of E_2_ (data not shown), whereas cellular T concentration remained unchanged (Fig.4B). There were significant group differences in the cellular concentration of E_2_ at all three time points (ANOVA *P *=* *0.001, 0.002 and < 0.001, respectively) following exposure to 100 nm E_2_. Cellular E_2_ concentration was significantly upregulated already at 6 h in 100 nm E_2_-exposed myotubes compared to mock (*P *=* *0.002) and continued to be elevated at 24 h (*P *=* *0.003) and at 72 h (*P *<* *0.001), while 10 nm E_2_ was not sufficient to increase the cellular concentration of E_2_ at any time point. However, there were no significant differences in the gene expression of *aromatase, ESR1*,*ESR2*,*GPER,* or *AR* at any of the time points after exposure to 10 or 100 nm E_2_ compared to the mock-treated cells (Fig.4C–G). Some uniform treatment-independent differences in the gene expression between time points were observed. There was a significant increment in the expression of *aromatase* at the 72-h time point compared to 6 and 24 h (ANOVA *P *<* *0.001). *ESR1* expression was lowest at the 24-h time point with all treatments (ANOVA *P *<* *0.001), and *ESR2* expression was highest at the 6-h time point with all treatments (ANOVA *P *<* *0.001).

### DHEA induces steroidogenesis in myotubes

**Figure 5:**
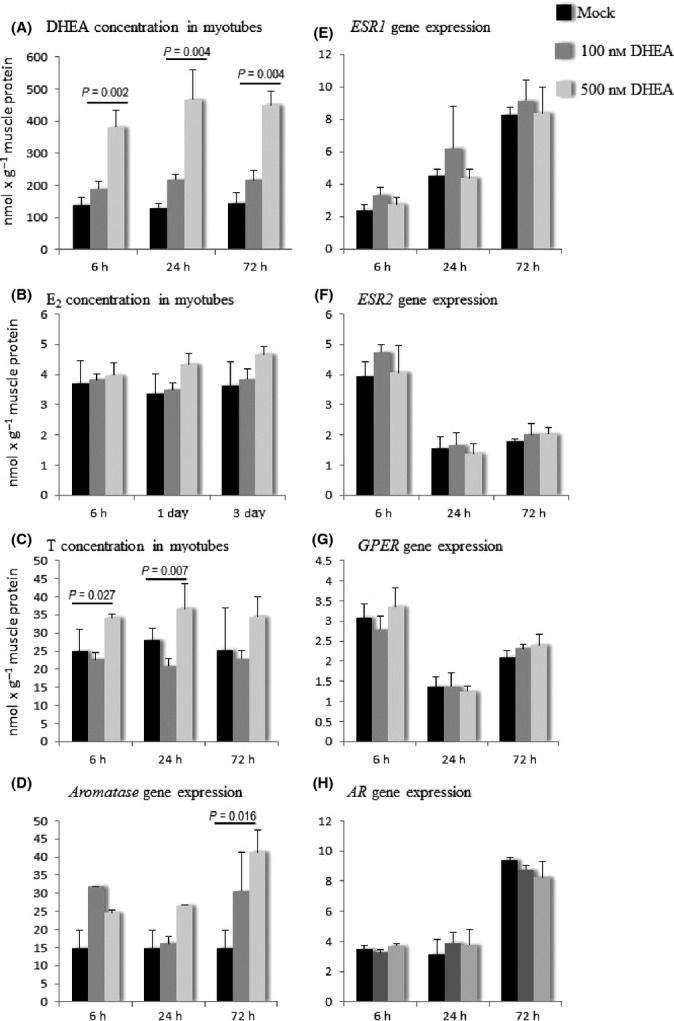
Sex steroid hormone concentration (A, B, C) and the gene expression of *aromatase* (D) and steroid hormone receptors (E, F, G, H) in myotubes exposed to DHEA. Gene expressions are normalized to the expression of *GAPDH* and presented in arbitrary units. DHEA, dehydroepiandrosterone; E_2_, 17β-estradiol; T, testosterone; *ESR1*, estrogen receptor 1; *ESR2*, estrogen receptor 2; *GPER*, G-protein-coupled estrogen receptor 1; *AR*, androgen receptor.

Finally, we examined whether the precursor hormone DHEA is taken up into the myotubes in culture and further processed to T or E_2_. After 5 days of differentiation, myotubes were exposed to 100 nm DHEA, 500 nm DHEA or mock for 6, 24 and 72 h. Myotubes readily took up DHEA into the cells as shown by the increase in the cellular concentration of DHEA (Fig.5A). There were significant group differences at all time points (ANOVA *P *=* *0.001, 0.003 and 0.002, respectively). Cellular DHEA concentration was significantly upregulated already at 6 h in 500 nm DHEA-exposed myotubes when compared to mock (*P *=* *0.002) and continued to be elevated at 24 h (*P *=* *0.004) and at 72 h (*P *=* *0.004), while 100 nm DHEA was not sufficient to increase cellular concentration of DHEA at any time point. Interestingly, the cellular concentrations of T and E_2_ also increased in response to the DHEA treatment, indicating that their synthesis from DHEA was induced. However, the differences in E_2_ concentration did not reach statistical significance (Fig.5B). Cellular T concentration was significantly different at 6- and 24-h time points, but not at 72 h (ANOVA *P *=* *0.007, 0.027 and 0.144, respectively; Fig.5C). In addition, the gene expression of aromatase was statistically significantly upregulated after 72-h exposure to 500 nm DHEA (*P *=* *0.13), while no significant differences in the gene expression of *ESR1*,*ESR2*,*GPER,* or *AR* were observed (Fig5D–H).

## Discussion

In the present study, we show that premenopausal women have larger thigh muscles with better performance (strength and power) than postmenopausal women although differences were more prominent when comparing to nonusers than to users of estrogen-containing HRT. This may be explained by protective actions of HRT, which elevate systemic E_2_ to levels closer to those observed in premenopausal women. However, the systemic steroid hormone levels were shown not to be related to intramuscular hormone levels. More importantly, intramuscular sex steroid hormones were strongly associated with muscle strength and power. From the measured intramuscular hormones E_2_, T, and DHEA were all shown to be independent predictors of knee extension strength and vertical jumping height. The final models adjusted for age, systemic E_2_, and body fat mass showed a highly significant association of intramuscular E_2_, T, DHT, and DHEA with muscle strength and power, but not with muscle size. This finding adds new insight into our current knowledge gained concerning the effects of systemic steroid hormones on aging-related changes in skeletal muscle properties in women (recently summarized in Sipilä *et al*., 2013; Tiidus *et al*., 2013). Previous studies have shown age-related reductions in systemic hormone concentrations to occur in parallel with the decline in muscle performance (Phillips *et al*., 1993) and that postmenopausal use of HRT is associated with better muscle performance (Sipilä *et al*., 2001; Ronkainen *et al*., 2009; Finni *et al*., 2011). Age-related muscle decline and the potential benefit from the use of HRT were also evident in the current study, but despite the age-related differences in systemic hormone concentrations, the intramuscular E_2_, T, and DHEA concentrations were similar in both pre- and postmenopausal women regardless of their HRT status. Only the intramuscular concentration of DHT was observed to be different between study groups, being higher in both postmenopausal groups as compared to the premenopausal women, which was completely opposite to what has been observed for systemic DHT concentration.

Intramuscular hormone levels were demonstrated to be significant predictors of muscle performance explaining roughly 60% of the variation in strength and 80% of the variation in power. This suggests that tissue-specific steroid hormones and their local synthesis may have functions which are independent from ovarian hormone secretion, which would regulate, for example, reproductive functions. For brain, the importance and independence of locally synthesized E_2_ has been recently highlighted (Li *et al*., 2014). It has been shown that brain, especially neurons, synthesizes E_2_ *de novo* from cholesterol, but circulating E_2_ or precursor hormones also penetrate through the blood–brain barrier (Kancheva *et al*., 2011). Therefore, the level of brain E_2_ may deviate from the systemic levels, and the source of brain hormones is not yet clear. Similar results have also been obtained for adipose tissue (Belanger *et al*., 2002). Prior to the current study, the intracrinology of skeletal muscle has been investigated only in a few rodent (Aizawa *et al*., 2007, 2008, 2010, 2011) and human studies (Vingren *et al*., 2008; Pöllänen *et al*., 2011; Sato *et al*., 2014a,b). These studies complemented by present study have clearly shown that skeletal muscle is able to take up sex steroid hormones and their precursors and is also able to locally synthesize biologically active hormones (T, E_2_, and DHT) from their precursors. However, the regulation of hormone uptake as well as the regulation and specific function of local, intramuscular steroidogenesis remains to be elucidated. So far, only the role of resistant exercise as a regulator of intramuscular steroidogenesis has been studied, but the results were inconclusive. Vingren *et al*. (2008) found no changes in intramuscular T synthesis after a single bout of resistance exercise in young male and female subjects, while Sato *et al*. found that 12 weeks of resistance training restored age-related differences in intramuscular DHEA and DHT in old male subjects (Sato *et al*., 2014a). Our previous study (Pöllänen *et al*., 2011) and the current one found no age-related decrement in intramuscular sex steroid hormones in female subjects over the studied age range. Contradictory results were found by Sato *et al*. (2014a) for older men whose intramuscular DHEA, free-T, and DHT levels were lower in old compared to young men, while in our current study, there were no differences in intramuscular DHEA, T, or E_2_ between old and young women, and higher intramuscular DHT was found in older than younger women. Without larger studies including both genders, we can only speculate whether this discrepancy is a true gender difference due to differences in hormonal aging of men and women. In addition to sex, there are also other differences in the study populations between these two studies. For example, our young group is in average over 10 years older and old group almost 10 years younger than in Sato's *et al*. study. Therefore, the age difference between groups in our study was approximately 26 years, while in Sato's *et al*. study, it was approximately 43 years. It would require longitudinal study or at least study using multiple age groups to be able to evaluate whether and how intramuscular hormone concentrations change during aging. In other words, we cannot be certain whether there is initial burst of increase in intramuscular steroidogenesis after cessation of ovarian steroidogenesis, which would later, with advancing age, decline. The other clear discrepancy between these two studies is the difference in the amount in total body fat between younger and older study groups. In our study, the fat mass of study participants was fairly similar between groups, while in Sato's *et al*. study, there was significant difference between young and old groups (body fat percentage 13.2% vs. 22.8%, *P* < 0.01). As adipocytes are also capable of steroid synthesis and it has been shown that increment in fat mass leads to decrement in the muscular expression of steroidogenic enzymes (Sato *et al*., 2014b), it cannot be ruled out that the higher amount of fat in older men would not decrease the demand for intramuscular steroidogenesis. Current study found intramuscular hormones to be associated with muscle strength and power in women, which is in line with results obtained for men (Sato *et al*., 2014a). However, we did not find the association between muscle cross-sectional area and intramuscular hormones in women as was found in men (Sato *et al*., 2014a).

Our results suggest that enzymes needed for local steroid synthesis in muscle must be regulated by other mechanisms than by systemic availability of hormones and that the uptake of systemic hormones to muscle cells is probably actively regulated. We were able to show that during *in vitro* differentiation of myoblasts into multinucleated myotubes, gene expression of *aromatase*,*ESR1*, and *AR* was induced, but that the cellular E_2_ concentration remains stable. The differences in the hormone concentrations in premenopausal serum, and postmenopausal nonusers’ or HRT users’ serum, did not affect the *in vitro* gene expression of *aromatase* or *AR*, but did change the gene expression of estrogen receptors *ESR1*,*ESR2,* and *GPER* in myotubes. The expression levels were higher in the myotubes exposed to the postmenopausal sera compared to premenopausal sera, suggesting that lower systemic E_2_ levels may induce transcription of the estrogen receptors. This could also, however, be due to other potential factors present in human sera. Therefore, to circumvent this caveat, we used pure E_2_ to see whether it is capable of mimicking the effects induced by female serum. The myotubes did take up E_2_, but it did not have any significant effect on gene expression. This finding further indicates that E_2_ does not directly regulate the transcription of *aromatase*, an enzyme required for the synthesis of E_2_ from its precursors or the transcription of steroid hormone receptors. However, it does show that myotubes respond to extracellular E_2_ and take it up, thereby indicating that some currently unknown factor in sera or in the cellular niche present *in vivo* regulates or inhibits spontaneous uptake of sex steroid hormones. Furthermore, the precursor hormone DHEA was also taken up into the myotubes which increased T and tended to increase the intracellular amount of E_2_, demonstrating that steroidogenesis was activated in myotubes. In addition, exposure to DHEA resulted in the transcriptional activation of *aromatase*, but not of the hormone receptors. Therefore, it still remains unclear as to how the transcription of steroid hormone receptors is regulated and how signaling mediated by these receptors is activated in skeletal muscle cells.

Skeletal muscle has recently been recognized as an endocrine organ communicating with other organs by secreting vast amounts of proteins and RNAs (Le Bihan *et al*., 2012). Currently, it is not known whether sex steroid hormones produced by muscle cells contribute to the secretome of muscle or function in an intra- or paracrine fashion within the muscle cells. A limitation of the current and previous studies regarding intramuscular steroidogenesis is the inability to identify the source of the hormones which are measured both in the muscle tissue and in the circulation. Therefore, we were not able to provide a clear consensus on the amount of hormones taken up by the skeletal muscle cells or the secretion of these hormones by the muscle fibers. In addition, we cannot confirm whether the flux of these hormones or their synthesis is affected by aging. Another limitation of the current study is the relatively small number of study participants. However, when invasive sampling such as muscle biopsies is required, the number of participants needs to be minimized to follow ethical recommendations in avoiding unnecessary discomfort and potential hazard caused to the participants. Limiting the number of the participants is often a trade-off between doing unnecessary invasive sampling and limiting the statistical power of a study. Retrospectively, we were successful in avoiding both pitfalls by finding highly significant associations between intramuscular sex steroid hormones and skeletal muscle strength and power. The discordant twin pair design is particularly suitable in this regard as the twins are matched on age, genomic sequence, and multiple other shared factors from childhood onwards, such that a small number of pairs have the statistical power of a much larger study on unrelated individuals. This is particularly true when the trait in question is under high genetic control, as evidenced by the high degree of similarity in intramuscular hormones in the MZ pairs.

Our results demonstrate that intramuscular sex steroids are involved in the regulation of muscle strength and power. We have also recently revealed the interaction between E_2_-responsive microRNAs (miR-182 and -223) and IGF-1/AKT pathway (Olivieri *et al*., 2014), which is major signaling pathway regulating muscle properties. The exposure of muscle cells to E_2_ was shown to increase the phosphorylation of AKT and mTOR, indicating higher activity of the pathway under higher E_2_ concentration. Therefore, microRNA-mediated regulation of IGF-1/AKT pathway is a potential molecular mechanism behind the intramuscular actions of steroid hormones. As a consequence, interventions aiming to manipulate intramuscular hormone synthesis may turn out to help to combat age-related muscle weakness. However, the field of muscle intracrinology is still in its infancy warranting further studies representing both genders with different age groups and physical activity levels to provide a consensus on the role and function of intracrine hormones in the regulation of muscle performance.

## Experimental procedures

### Human study design and phenotype measurement

This study uses a subsample of a larger SAWEs Study (Ronkainen *et al*., 2009; Pöllänen *et al*., 2011) including postmenopausal HRT discordant MZ twin pairs and a cohort of premenopausal women with no hormonal treatment. Postmenopausal women were recruited from the Finnish Twin cohort (Kaprio & Koskenvuo, 2002). From the HRT discordant MZ twin pairs recruited to SAWEs, the current study utilizes data and samples of eight pairs from whom one other sister was current user of estrogen-containing HRT, while the other had never used HRT. The median and interquartile range of the duration of the HRT was 6 ± 5 years. Eight total body fat-matched premenopausal women were selected from the age cohort of premenopausal women included in SAWEs. Matching for body adiposity between groups was carried out to avoid the possible influence of differences in the amount of adipogenic steroidogenesis on the systemic or muscle specific effects under investigation. Fat and lean body mass were measured with the multifrequency bioelectrical impedance analyzer (InBody 720; Biospace, Seoul, Korea). Muscle strength, power, and size were assessed as reported in Ronkainen *et al*. (2009). Briefly, isometric knee extension strength was assessed with an adjustable dynamometer chair (Good Strength; Metitur, Palokka, Finland). The explosive lower body muscle power, that is, ability to produce force as quickly as possible, was assessed as the maximum height that the participant was able to elevate her body's center of gravity during a vertical jump on a contact mat. In all measurements, three to five trials were allowed and the maximal performance was accepted as the result. Mid-thigh muscle CSA was obtained using computed tomography scanner (Siemens Somatom Emotion scanner; Siemens, Erlangen, Germany) taken from the midpoint between the greater trochanter and the lateral joint line of the knee. Fat infiltrated into the muscle compartment was excluded from the analysis. Physical activity volume was retrospectively inferred using standard questionnaire concerning previous 12 months and seven consecutive days including leisure-time physical activity, daily activities such as gardening, and commute physical activity from which the mean total daily metabolic equivalent (MET) index was calculated as MET-h per day (Finni *et al*., 2009). This study was approved by the Local Ethical Committee of the Central Finland Hospital District (decision # E0606/06) and performed following guidelines of the Helsinki declaration and good clinical and scientific practice. All study participants signed an informed consent form.

### Serum and muscle sampling

Blood samples were taken between 07:00 and 09:00 h in standard fasted conditions with participants in supine position. Sera were stored at −80 °C until analysis. Directly following blood sampling, muscle biopsies were taken from the mid-portion of the *m. vastus lateralis* from all study participants using a standardized protocol. The blood samples of premenopausal women were collected during 1–5 days of estrous cycle. Visible blood and fat were removed before the biopsy sample was snap-frozen in liquid nitrogen and stored at −80 °C until used for mRNA, protein, or hormone analysis.

For the cell culture experiments, equal amounts of each serum sample were pooled together to form either premenopausal serum, postmenopausal serum with HRT (HRT users), or postmenopausal serum without HRT (HRT nonusers).

### Systemic and intramuscular hormone measurements

Serum and muscle hormone assessments were carried out using the protocols presented in Pöllänen *et al*. (2011). Briefly, serum concentrations of sex hormone-binding globulin (SHBG), DHEAS, follicle-stimulating hormone (FSH), and luteinizing hormone (LH) were measured using solid-phase, chemiluminescent immunometric assays (Immulite 1000; Diagnostic Products, Los Angeles, CA, USA). Serum E_2_ was determined using an extraction radioimmunoassay, while T and DHT were measured separately using LC-MS/MS. Muscle samples were homogenized on ice in Tissue Extraction Reagent I (Invitrogen, Carlsbad, CA, USA) supplemented with protease and phosphatase inhibitors. The amount of total soluble protein was determined using Pierce BCA Protein Assay kit (Thermo Scientific, Rockford, IL, USA). Elisa tests were used to determine E_2_, T, DHT, and DHEA concentrations in 1:10 diluted muscle supernatants in duplicates (IBL International, Hamburg, Germany). The concentrations of intramuscular hormones were expressed as nmol g^−1^ soluble muscle protein.

### Cell culture experiments

Cell culture medium, fetal bovine serum (FBS), and antibiotics were obtained from Life Technologies, Inc. (Carlsbad, CA, United States). Phenol Red free DMEM was used in all experiments. Insulin and apotransferrin were purchased from Sigma-Aldrich (St. Louis, MO, USA). Primary human muscle cell line was derived from a quadriceps muscle biopsy of a 5-day-old female infant, in accordance with the French legislation (Edom *et al*., 1994). The cells were maintained as proliferating mononuclear myoblasts by cultivation in 4:1 DMEM:199 medium supplemented with 20% FBS, 1% sodium pyruvate, 100 U mL^−1^ penicillin, and 100 μg mL^−1^ streptomycin. To induce differentiation into multinuclear myotubes, myoblasts were cultured in differentiation medium containing 1:4 DMEM:199 supplemented with 1% sodium pyruvate, 10 μg mL^−1^ insulin, 100 μg mL^−1^ apotransferrin, 10 U mL^−1^ penicillin, and 10 μg mL^−1^ streptomycin. To study the expression of steroid hormone receptors (*ESR1*,*ESR2*,*GPER,* and *AR*) and *aromatase* (*CYP19A1* gene) during the differentiation of myoblasts into myotubes, the cells were harvested for RNA isolation either just before switching to the differentiation medium (0 day sample) or after every 24 h for 7 days (1–7 days samples). Three independent samples were prepared for each time point. To investigate the effects of pre- and postmenopausal serum with their inherent hormone status on steroid hormone signaling, myotubes at day five were exposed to the serum at final concentration of 10% v/v of differentiation medium. Each serum pool contained equal amount of sera from eight participants per group. Each exposure was performed in triplicates, and exposed cells were collected for RNA isolation at 6-, 24-, and 72-h time points after exposure. To investigate the effects of synthetic DHEA and E_2_, myotubes differentiated for 5 days were exposed to 100 or 500 nm DHEA, 10 or 100 nm E_2_, or mock for 6, 24, and 72 h before RNA isolation. Mock was differentiation medium supplemented with ethanol, which was used as solvent for DHEA and E_2_. All experiments were carried out in triplicate.

### Quantitative PCR

NORGEN RNA kit (Miliot Science, Porvoo, Finland) was used to isolate total RNA from cultured cells. RNA concentration was determined by absorbance at 260 nm (NanoDrop ND-1000; Thermo Fisher Scientific Inc., Waltham, MA, USA), and its purity was assessed based on a 260/280 absorbance ratio of ∽2.0. High-capacity cDNA Reverse Transcription kit was used for cDNA synthesis (Applied Biosystems, Foster City, CA, USA). Quantitative real-time PCR (qPCR) was performed according to Pöllänen *et al*. (2011).

### Statistics

Results are presented as mean ± standard deviation (SD) or median and interquartile range (25th–75th percentile). Analyses were conducted using stata 13.0 (College Station, TX, USA). Statistical testing based on individuals was conducted using methods (*svy* procedures) taking into account the lack of statistical independence of twin sisters. The adjusted Wald test or ANOVA followed by Tukey's post hoc test were used to test the significance of the differences between study groups and cell exposures, respectively. Pearson's correlation coefficient (*r*) and intraclass correlation were calculated as a measure of within-pair similarity between co-twins. Because MZ twin pairs are genetically identical, any observed intrapair difference by definition is due to individual environmental effects. Therefore, *r* can be taken as an estimate of the magnitude of genetic and environmental effects on tested variables. Multivariate linear regression models were constructed to estimate the association between intramuscular steroid hormones and muscle strength, power, and cross-sectional area. It is known that muscle variables are affected also by age, systemic E_2_, physical activity, and potentially nutrition. In addition, adipose tissue is the potential source of steroid hormones and may thereby affect intramuscular steroidogenesis. Unfortunately, we did not have nutritional data available from the premenopausal group. The association of these other potential confounders with muscle strength, power, and cross-sectional area was investigated by bivariate linear regression to estimate the magnitude of the association, and only the variables showing some independent significant association with the muscle performance variables (Table S2, Supporting information) were included step-by-step into the final models (Tables3 and S1). Model 1 is a bivariate model showing the association between the intramuscular hormone concentration and muscle performance variable. Models 2–4 are multivariate models including intramuscular hormone concentration and age, systemic E_2_ concentration, and total body fat mass incorporated one at the time. *R*^2^ shows coefficient of determination. The level of significance was set at *P *≤* *0.05.

## Supporting Information

**Table S1** Linear regression models for skeletal muscle size (Thigh muscle cross-sectional area). **Table S2** Bivariate linear regression models for association of potential confounders with muscle strength, power and size.
