# Supplementary material for: Intramuscular sex steroid hormones are associated with skeletal muscle strength and power in women with different hormonal status
Source: Aging Cell. 2015 Jan 20;14(2):236–48. doi: 10.1111/acel.12309 (PMC4364836; doi:10.1111/acel.12309)
Supplement: Supplementary file 1 [file acel0014-0236-sd1.docx]

**Supporting Information**

**Table S1.** Linear regression models for skeletal muscle size (Thigh muscle cross-sectional area).

|  | **Model 1**  **B (standard error)** | | | **Model 2**  **B (standard error)** | **Model 3**  **B (standard error)** | **Model 4**  **B (standard error)** |
| --- | --- | --- | --- | --- | --- | --- |
| ***Estrogen models*** | | | | | | |
| Intramuscular E_2_ | 763.7 (773.5)  P=0.340 | | | 94.1 (701.7)  P=0.895 | -37.1 (626.2)  P=0.954 | 389.5 (363.3)  P=0.302 |
| Age |  | | | -45.5 (22.8)  P=0.066 | -25.9 (24.7)  P=0.312 | -20.4 (24.7)  P=0.423 |
| Systemic E_2_ |  | | |  | 1.7 (1.1)  P=0.156 | 2.5 (1.1)  P=0.042 |
| Fat mass |  | | |  |  | 73.2 (30.7)  P=0.032 |
| *R^2^ for a model* | *0.033* | | | *0.176* | *0.249* | *0.387* |
| ***Testosterone models*** | | | | | | |
| Intramuscular T | 80.7 (76.1)  P=0.307 | | | 20.6 (69.4)  P=0.771 | 11.5 (61.0)  P=0.853 | 35.9 (44.7)  P=0.435 |
| Age |  | | | -44.8 (22.7)  P=0.068 | -24.7 (24.6)  P=0.331 | -21.2 (24.8)  P=0.409 |
| Systemic E_2_ |  | | |  | 1.7 (1.1)  P=0.160 | 2.5 (1.1)  P=0.043 |
| Fat mass |  | | |  |  | 71.3 (31.8)  P=0.042 |
| *R^2^ for a model* | *0.033* | | | *0.178* | *0.250* | *0.387* |
| ***DHT models*** | | | | | | |
| Intramuscular DHT | | -1510.4 (1346.9)  P=0.281 | | 328.5 (1282.8)  P=0.802 | 323.3 (1168.3)  P=0.786 | 691.9 (1007.6)  P=0.504 |
| Age | |  | | -49.4 (26.8)  P=0.087 | -28.4 (26.6)  P=0.303 | -29.8 (25.5)  P=0.262 |
| Systemic E_2_ | |  | |  | 1.7 (1.2)  P=0.163 | 2.5 (1.1)  P=0.042 |
| Fat mass | |  | |  |  | 71.0 (31.7)  P=0.042 |
| *R^2^ for a model* | | *0.044* | | *0.177* | *0.250* | *0.388* |
| ***DHEA models*** | | | | | | |
| Intramuscular DHEA | | | 20.1 (13.2)  P=0.151 | 9.6 (12.7)  P=0.460 | 7.8 (9.8)  P=0.436 | 9.4 (7.9)  P=0.251 |
| Age | | |  | -41.4 (23.6)  P=0.101 | -22.0 (25.0)  P=0.392 | -19.5 (25.9)  P=0.464 |
| Systemic E_2_ | | |  |  | 1.7 (1.1)  P=0.161 | 2.4 (1.1)  P=0.042 |
| Fat mass | | |  |  |  | 70.4 (32.1)  P=0.046 |
| *R^2^ for a model* | *0.069* | | | *0.190* | *0.258* | *0.395* |

**Table S2.** Bivariate linear regression models for association of potential confounders with muscle strength, power and size.

|  | **Model for physical activity**  **B (standard error)** | | **Model for age**  **B (standard error)** | **Model**  **for systemic E_2_**  **B (standard error)** | **Model for fat mass**  **B (standard error)** |  |  |  |  |
| --- | --- | --- | --- | --- | --- | --- | --- | --- | --- |
|  | | | | | |  |  |  |  |
| ***Association with muscle strength*** | | 3.7 (3.8)  P=0.338 | -5.5 (1.7)  **P=0.005** | 0.16 (0.06)  **P=0.024** | 3.6 (2.8)  P=0.209 |  |  |  |  |
| *R^2^ for a model* | | *0.035* | *0.376* | *0.161* | *0.071* |  |  |  |  |
|  | |  |  |  |  |  |  |  |  |
| ***Association with muscle power*** | | 0.1 (0.3)  P=0.679 | -0.4 (0.1)  **P<0.001** | 0.01 (0.01)  **P=0.002** | -0.36 (0.2)  **P=0.047** |  |  |  |  |
| *R^2^ for a model* | | *0.007* | *0.547* | *0.340* | *0.169* |  |  |  |  |
|  | | | | | |  |  |  |  |
| ***Association with muscle size*** | | 43.8 (47.4)  P=0.370 | -46.3 (20.8)  **P=0.042** | 2.15 (0.84)  **P=0.022** | 36.1 (31.0)  P=0.263 |  |  |  |  |
| *R^2^ for a model* | | *0.034* | *0.192* | *0.219* | *0.050* |  |  |  |  |
